# Insights into the Recent 2019 Novel Coronavirus (SARS-CoV-2) in Light of Past Human Coronavirus Outbreaks

**DOI:** 10.3390/pathogens9030186

**Published:** 2020-03-04

**Authors:** Hossam M. Ashour, Walid F. Elkhatib, Md. Masudur Rahman, Hatem A. Elshabrawy

**Affiliations:** 1 Department of Biological Sciences, College of Arts and Sciences, University of South Florida St. Petersburg, St. Petersburg, FL 33701, USA; 2 Department of Microbiology and Immunology, Faculty of Pharmacy, Cairo University, Cairo 11562, Egypt; 3 Department of Microbiology and Immunology, School of Pharmacy & Pharmaceutical Industries, Badr University in Cairo (BUC), Entertainment Area, Badr City, Cairo 11829, Egypt; walid2005faisal@yahoo.com; 4 Microbiology and Immunology Department, Faculty of Pharmacy, Ain Shams University, African Union Organization St., Abbassia, Cairo 11566, Egypt; 5 Department of Pathology, Faculty of Veterinary, Animal and Biomedical Sciences, Sylhet Agricultural University, Sylhet 3100, Bangladesh; rahmanmm.dpp@sau.ac.bd; 6 Department of Molecular and Cellular Biology, College of Osteopathic Medicine, Sam Houston State University, Conroe, TX 77304, USA

**Keywords:** 2019-nCoV, SARS-CoV-2, SARS, MERS, COVID-19

## Abstract

Coronaviruses (CoVs) are RNA viruses that have become a major public health concern since the Severe Acute Respiratory Syndrome-CoV (SARS-CoV) outbreak in 2002. The continuous evolution of coronaviruses was further highlighted with the emergence of the Middle East Respiratory Syndrome-CoV (MERS-CoV) outbreak in 2012. Currently, the world is concerned about the 2019 novel CoV (SARS-CoV-2) that was initially identified in the city of Wuhan, China in December 2019. Patients presented with severe viral pneumonia and respiratory illness. The number of cases has been mounting since then. As of late February 2020, tens of thousands of cases and several thousand deaths have been reported in China alone, in addition to thousands of cases in other countries. Although the fatality rate of SARS-CoV-2 is currently lower than SARS-CoV, the virus seems to be highly contagious based on the number of infected cases to date. In this review, we discuss structure, genome organization, entry of CoVs into target cells, and provide insights into past and present outbreaks. The future of human CoV outbreaks will not only depend on how the viruses will evolve, but will also depend on how we develop efficient prevention and treatment strategies to deal with this continuous threat.

## 1. Introduction

**Figure 1:**
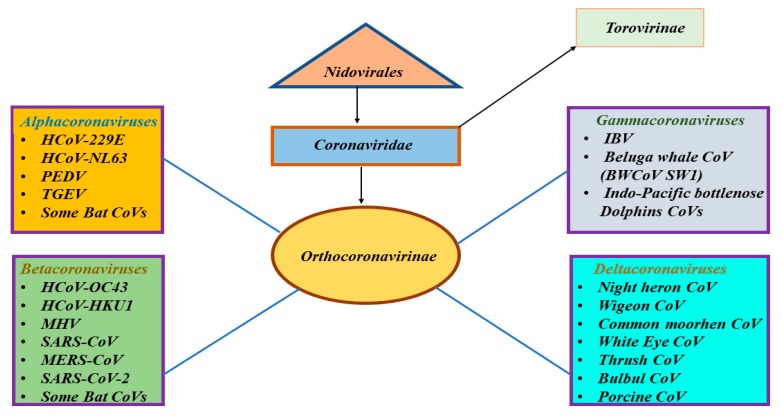
Classification of different types of coronaviruses within the family *Coronaviridae,* subfamily *Orthocoronavirinae,* and the respective genera: alpha-, beta-, gamma-, and deltacoronaviruses. The SARS-CoV-2 is classified as a betacoronavirus.

Coronaviruses (CoVs) were discovered in the 1960s and they were classified under family *Coronaviridae*, which is the largest family within the order *Nidovirales* (Figure 1) [1]. Family *Coronaviridae* encompasses two subfamilies: subfamily *Orthocoronavirinae* and subfamily *Torovirinae* (Figure 1) [1]. Subfamily *Orthocoronavirinae* includes four genera: alphacoronavirus, betacoronavirus, gammacoronavirus, and deltacoronavirus (Figure 1) [1]. CoVs are typically harbored in mammals and birds and are common in camels, cattle, cats, bats, and other animals [2]. Alpha and betacoronaviruses circulate in mammals, including bats (Figure 1) [2]. Gammacoronaviruses mostly infect avian species and a few mammalian species, whereas deltacoronaviruses infect birds and mammals (Figure 1) [2]. Animal CoVs are known to cause important diseases in animals and could be responsible for economic losses in domestic animals or birds [3,4,5]. These animal CoVs include avian infectious bronchitis virus (IBV), transmissible gastroenteritis virus (TGEV), porcine epidemic diarrhea virus (PEDV), and more recently, swine acute diarrhea syndrome-CoV (SADS-CoV). Although rare, animal CoVs have the ability to infect humans and could further spread through human-to-human transmission [6,7].

The first discovered CoVs were IBV that causes respiratory disease in chickens and the human CoVs, human CoV-229E (HCoV-229E) and human CoV-OC43 (HCoV-OC43), which cause the common cold in humans [8,9]. Since the emergence of HCoV-229E and HCoV-OC43, several other HCoVs were discovered, such as Severe Acute Respiratory Syndrome-CoV (SARS-CoV) in 2002, HCoV-NL63 in 2004, HCoV-HKU1 in 2005, Middle East Respiratory Syndrome-CoV (MERS-CoV) in 2012 [10]. Starting December 2019, there were reports of patients presenting with severe viral pneumonia in the city of Wuhan, China [11]. Sequencing of the virus from these patients has identified a novel CoV as the causative agent of this respiratory disease [11]. The 2019 novel CoV virus (2019-nCoV) was recently named SARS-CoV-2 by the World Health Organization (WHO). The disease caused by SARS-CoV-2 has been named COVID-19. Prior to 2002, CoVs were treated as nuisances but never as serious viruses. Things changed after the emergence of SARS-CoV, which caused serious illnesses and deaths in 2002–2003 [12]. Unlike all human CoVs that cause mild respiratory symptoms, SARS-CoV, MERS-CoV, and SARS-CoV-2 are associated with serious respiratory diseases [12,13]. Since its emergence, the SARS-CoV-2 has drawn well-deserved attention from the world. Efforts are underway in an attempt to control this new CoV outbreak.

## 2. Coronavirus Structure

CoVs, including the newly discovered SARS-CoV-2, are spherical positive single-stranded RNA viruses that are characterized by spike proteins projecting from the virion surface [14,15]. The spherical morphology of the viral particle together with the spike projections led to the name coronavirus from the Latin word corona meaning crown, due to the appearance of the virus as a royal crown under the electron microscope [14,15]. CoVs are enveloped viruses (envelope is a lipid bilayer derived from the host cell membrane) with the viral structure formed primarily of structural proteins such as spike (S), membrane (M), envelope (E), and nucleocapsid (N) proteins, and hemagglutinin-esterase (HE) protein in some betacoronaviruses [16]. The S, M, and E proteins are all embedded in the viral envelope; however, N protein interacts with the viral RNA and is located in the core of the viral particle, forming the nucleocapsid [16]. The S protein is a heavily glycosylated protein that forms homotrimeric spikes on the surface of the viral particle and mediates viral entry into host cells [17]. In some CoVs, each monomer of the homotimeric S protein exists as two subunits (S1 and S2) on the viral particle due to cleavage of S protein by host furin-like proteases during viral replication [17,18]. However, in other CoVs including SARS-CoV, S protein forms S1 and S2 domains, remains intact on viral particles, and only gets cleaved inside endocytic vesicles during viral entry [19,20].

The M protein is one of the most important proteins in the virion structure. It exists in higher quantities than any other protein in the viral particle, in contrast to the E protein which is found in small quantities within the virion [21]. The difference in abundancy may due to the fact that M protein gives the virus its shape and is critical together with E protein in orchestrating the assembly of the virus and in forming mature viral envelopes [22]. The E protein also functions in release of viral particles from host cells, in addition to other functions [22].

The N protein binds the viral RNA and is required for packaging of viral RNA into the viral particle during viral assembly [23,24]. As mentioned previously, HE is present on the surface of some betacoronaviruses. It is a hemagglutinin similar to influenza virus hemagglutinin (binds sialic acid on host cell-surface glycoproteins) and possesses acetyl-esterase activity [25]. HE characteristics may enhance entry and pathogenesis of coronaviruses that contain such protein in their viral structure.

## 3. Genome Organization and Replication

The RNA genome of CoVs is the second largest of all RNA viruses, ranging from 26 to 32 kilobases (kb) in size [26]. The largest genome of all RNA viruses is that of the recently described planarian secretory cell nidovirus, PSCNV (41.1 kb genome size) [27]. Viral RNA codes for structural and nonstructural proteins [28]. The structural proteins together with a few nonstructural proteins, with different functions, are coded within the 3′ end of the viral genome [28]. However, the 5’ two-thirds of the genome codes for nonstructural proteins that are important in viral replication, including the RNA-dependent RNA polymerase (RdRP) [28]. Once the viral genome is inside the host cell cytoplasm following viral entry, translation of the 5′ end of viral RNA produces the RdRP, which uses viral RNA as a template to generate virus-specific mRNAs (subgenomic mRNAs) from subgenomic negative strand intermediates [29,30]. Subgenomic mRNAs share the same 3′ ends and the same leader sequence of 70–90 nucleotides at their 5′ ends [28,31]. Translation of subgenomic mRNAs leads to production of structural and nonstructural viral proteins [28]. Once sufficient structural proteins and genomic viral RNA are formed, viral RNA is then assembled with viral structural proteins into virions. Viral assembly and budding occur in smooth-walled vesicles in the endoplasmic reticulum–Golgi intermediate compartment (ERGIC) [28].

## 4. Coronavirus Entry

S protein is the viral protein that mediates the entry of CoVs into host cells [32]. Receptor-binding domain (RBD) within the S1 domain mediates binding to the cognate host cell receptor; however, the S2 domain mediates the fusion events, between viral membrane and host cell membrane, that are required for entry of CoVs into host cells [33,34].

The RBD is located at N-terminus of the S1 subunit as in mouse hepatitis virus (MHV) or at the C- terminus as in SARS-CoV and MERS-CoV [34,35]. The tissue tropism of CoVs is determined by the S protein interaction with the receptors on host cells. Several cellular receptors were described as receptors for CoVs. For example, aminopeptidase N (APN) was identified as the receptor for several alphacoronaviruses [36], angiotensin-converting enzyme 2 (ACE2) as the receptor for SARS-CoV [37], HCoV- NL63 [38] and possibly for the newly discovered SARS-CoV-2 [39], CEACAM1 as the receptor for MHV [40], and dipeptidyl-peptidase 4 (DPP4 which is also known as CD26) as the receptor for MERS-CoV [41].

A study on the RBD of SARS-CoV-2 S protein showed its similarity in structure to that of SARS-CoV with some key amino acid differences [42]. The previous finding suggests that SARS-CoV-2 could employ human ACE2 as its cellular receptor. A recent study published in Science showed that SARS-CoV-2 S protein has higher affinity to ACE2 than SARS-CoV S protein [43]. However, the receptor usage and the cellular tropism of SARS-CoV-2 need further investigation.

The trimeric CoV S protein is cleaved by host cell proteases during infection to expose the fusion peptide of the S2 domain, which induces the fusion of viral and cellular membranes [18,20,44,45]. Fusion of viral envelope with host cell membrane results in the release of the viral genome into the cytoplasm [33,34]. Cleavage of S protein occurs at different sites that were identified, in different coronaviruses, to be between the S1 and S2 domains (S1/S2 site) and within the S2 domain proximal to the fusion peptide (S2′ site) [18,20,44,45]. It is believed that cleavage at both sites is required for viral entry [46]. Different proteases have been identified to cleave the S1/S2 site depending on its amino acid sequence in different viruses. For example, the S1/S2 site of MERS-CoV S protein (RSVR↓SV) is cleaved by furin after its biosynthesis during viral replication [47]. SARS-CoV-2 has an S1/S2 site (AYT↓M) that is identical to the one in SARS-CoV [48]. This SARS-CoV S1/S2 site has been shown to be cleaved by cathepsin L following receptor binding and during viral entry in late endosomes [20]. We believe that the SARS-CoV-2 S1/S2 site may be cleaved by cathepsin L similar to SARS-CoV. Other proteases such as trypsin, elastase, and TMPRSS2 have been shown to cleave SARS-CoV S protein at other sites between S1 and S2 domains [44,46,49]. Unlike SARS-CoV, SARS-CoV-2 has an additional furin-like protease cleavage site (RRAR↓SV) that is N-terminus to the S1/S2 site (AYT↓M), and is absent in SARS-CoV [48]. The presence of this furin-like cleavage site in SARS-CoV-2 suggests its cleavage by furin during viral egress. In addition to the previous S1/S2 sites, SARS-CoV-2 has a furin-like protease cleavage S2′ site (KR↓SF) that is identical to that in SARS-CoV [48]. However, there is no evidence that SARS-CoV S protein is cleaved by furin-like proteases at the S2′ site during viral egress [20]. Similar to S1/S2 site, we believe that SARS-CoVS protein is cleaved at the S2′ site by cathepsin L or TMPRSS2 in different cellular locations during viral entry. The previous notion is supported by several studies which showed that cathepsin L and TMPRSS2 promote SARS-CoV entry while their inhibition suppressed infection of permissive cells [20,44,50]. Given that SARS-CoV-2 has the same S2′ site as SARS-CoV, we believe that processing of SARS-CoV-2 S protein at S2′ site is similar to SARS-CoV. Similarly, MERS-CoV has an S2′ site (RXXR↓SA) that is less efficiently cleaved by furin and most probably cleaved by TMPRSS2 or cathepsin L during viral entry [51]. Despite the identification of putative protease cleavage sites in SARS-CoV-2 S protein, their relative importance for SARS-CoV-2 S protein activation, viral pathogenesis, and cellular tropism needs further investigation. Antiviral small molecules that inhibited cathepsin L were able to inhibit SARS-CoV infections in vitro and that of other viruses that depend on cathepsin L for entry, such as Ebola, Hendra, and Nipah viruses [50]. The presence of a cathepsin L cleavage site in SARS-CoV-2 S protein (S1/S2 site) suggests that cathepsin L inhibitors may be valuable in inhibiting SARS-CoV-2 infections [48]. Antibodies against RBD and S2 domain of SARS-CoV and MERS-CoV S proteins have been found effective in neutralizing infections of permissive cell lines in vitro [52,53,54,55]. In addition, neutralizing antibodies were capable of treating infections in experimental animals and in infected patients during these major outbreaks [56,57,58,59]. In one study, several SARS-CoV RBD-specific monoclonal antibodies did not bind to SARS-CoV-2 S protein [43]. Another study showed that the SARS-CoV-specific monoclonal antibody, CR3022, bound with high affinity to SARS-CoV-2 RBD [60]. The previous studies suggest that there are differences between the two RBDs which impact the cross reactivity of many neutralizing antibodies. However, neutralizing antibodies against SARS-CoV-2, once developed, could be promising in controlling the current SARS-CoV-2 outbreak.

## 5. SARS-CoV, MERS-CoV, and the Newly Discovered SARS-CoV-2: Similarities and Differences

**Figure 2:**
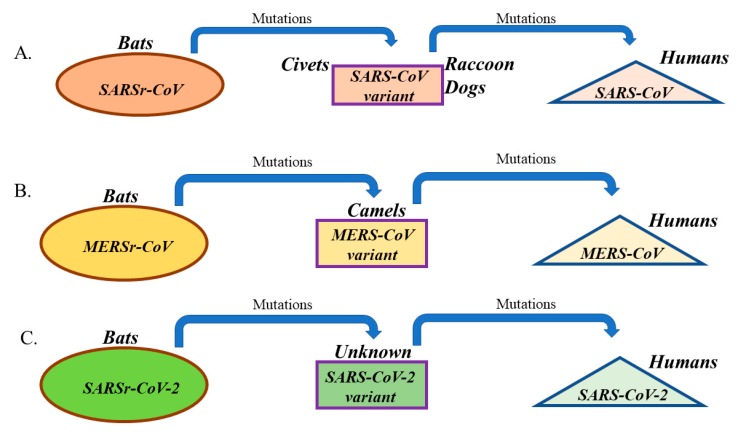
Origin and evolution of (**A**) SARS-CoV, (**B**) MERS-CoV, and (**C**) SARS-CoV-2 in different hosts. All viruses initially existed in bats as CoV-related viruses (SARSr-CoV, MERSr-CoV, and SARSr-CoV-2) before acquiring mutations and adapting to intermediate hosts and ultimately humans.

SARS-CoV was identified as a human CoV that causes severe acute respiratory syndrome (SARS) in the 2002–2003 outbreak that occurred in Guandong province, China and resulted in 774 deaths out of around 8098 cases that were infected over nine months (around 10% fatality) (Table 1) [12]. SARS-CoV was found to infect unciliated bronchial epithelial cells and type II pneumocytes and cause fever, cough, shortness of breath, and severe complications such as pneumonia and kidney failure [12,37]. The incubation period for SARS-CoV was estimated to range from 2 to 10 days, and up to 14 days (Table 1) [61]. Studies have shown that bats harbor CoVs that are ancestral to SARS-CoV (Table 1) [62]. Civets and raccoon dogs, of Chinese local markets, were shown to harbor SARS-like CoVs (Table 1) [63]. The detection of SARS-related CoVs (SARSr-CoVs) in bats and small animals in retail markets may indicate an interspecies transmission from bats to small animals and finally to humans (Figure 2). Studies in bats from different regions of China have identified several SARSr-CoVs [64]. The previous finding indicates that SARS-CoV has been circulating in bats for a long time before genetically changing and jumping to humans. Since ACE2 was identified as the receptor for SARS-CoV, it is not surprising that SARS-CoV has adapted itself to bind human ACE2 and efficiently infect human cells [65]. That sort of adaptation required a set of amino acid changes in the RBD of S protein of SARS viruses that were circulating in bats [65]. Therefore, we conclude that the human-to-human transmission that was seen during the SARS-CoV outbreak is attributed to the ability of SARS-CoV to adapt its S protein (particularly RBD) to efficiently bind to human ACE2 and infect airway epithelia (Figure 2).

MERS-CoV was first described in 2012 as a new CoV that causes a severe respiratory disease in Saudi Arabia [13]. Similar to SARS-CoV, MERS-CoV infects unciliated bronchial epithelial cells and type II pneumocytes and causes severe illness of the respiratory tract, which is characterized by fever, cough, shortness of breath, and severe complications such as pneumonia and kidney failure [13]. The incubation period of MERS-CoV is quite similar to SARS-CoV and ranges from 2 to 14 days (Table 1) [66]. As of January 2020 and since 2012, 862 of 2506 infected cases in 27 countries have died (≈35% fatality), which is more than three times the fatality seen in SARS-CoV infections (Table 1) [67]. However, unlike SARS-CoV, human-to-human transmission of MERS-CoV is not easy and has not been confirmed except in cases of very close contact with infected patients in health care settings [67]. MERS-related CoVs (MERSr-CoVs) were detected in bats, suggesting a potential bat origin (Table 1) [68,69]. MERS-CoV was transmitted to humans from dromedary camels (Table 1) [70]. Studies have also shown that camel MERS-CoV strains are almost identical to human MERS-CoV strains [71]. It was postulated that MERS-CoV existed in camels at least 30 years ago since antibodies to MERS-CoV were detected in samples that were collected from camels in 1983 [72]. Sequence analyses have shown that MERSr-CoVs’ RBDs share only 60–70% sequence identity with that of human and camel MERS-CoVs [73]. Similar to the adaptation of SARS-CoV to human host, MERSr-CoVs that are circulating in bats had to undergo several amino acid changes in RBD of S protein to become capable of infecting camels and humans (Figure 2) [74]. We believe that the amino acid changes in MERSr-CoVs’ RBD led to the emergence of MERS-CoV strains that are capable of binding to human DPP4 with high affinity, infecting humans, and causing the 2012 outbreak (Figure 2).

As mentioned previously, the SARS-CoV-2 was isolated and sequenced from patients that showed symptoms of respiratory illness and pneumonia in Wuhan, China during December 2019. SARS-CoV-2 is the third identified human CoV that causes severe respiratory illness with symptoms and incubation period resembling that of SARS-CoV and MERS-CoV infections (Table 1) [11,75]. Since December 2019, SARS-CoV-2 infection rates have been rising in China and worldwide [42]. Similar to SARS-CoV and unlike MERS-CoV, human-to-human transmission has been confirmed [42]. Initial cases of SARS-CoV-2 infections were somehow connected to the Huanan Seafood Market in Wuhan, in the Hubei province of China [11]. In this market, a number of nonaquatic animals were on sale, such as birds, snakes, marmots, bats, and rabbits [11]. Genetic analyses of viral samples from patients with SARS-CoV-2 infections revealed that the SARS-CoV-2 is a betacoronavirus that has 88% sequence identity to two bat SARSr-CoV: 79% identity to SARS-CoV and only 50% identity to MERS-CoV [42]. The previous findings suggest that SARS-CoV-2 is a new virus that is distinct from SARS-CoV and MERS-CoV but most probably originated in bats, similar to SARS-CoV and MERS-CoV [42]. Another recent study confirmed that SARS-CoV-2 significantly clustered with a sequence from the bat SARS-like CoV that was isolated in 2015 [76]. However, the existence of an intermediate host for SARS-CoV-2 is still not verified (Figure 2).

To date, the fatality of SARS-CoV-2 appears to be less than that observed in SARS-CoV and MERS-CoV infections. However, since new cases are confirmed everyday as we write this review, the fatality of this virus may keep changing and will not be accurately calculated until after the end of this outbreak. The virus appears to be more fatal in elderly patients or patients with comorbidities [77]. However, it is important to note that there could be cases that went undetected, which makes it hard to accurately calculate the fatality of this new virus.

## 6. Insights and Lessons Learned from SARS-CoV, MERS-CoV, and SARS-CoV-2 Outbreaks

Our lessons learned from SARS-CoV and MERS-CoV outbreaks include the high mutation rates that characterize all RNA viruses [78], the evolving nature of CoVs [6,7], and the ease of transmission from one species to another [6,7]. As mentioned previously, it appears that SARS- and MERS-CoVs arose at sometime from ancestral CoVs harbored by bats (Figure 2). Whereas animals served as intermediate hosts, humans served as terminal hosts (Figure 2). SARS-CoV was transmitted to civets and raccoon dogs, and to camels in the case of MERS-CoV (Figure 2). They were then transmitted from these intermediate animal hosts to humans (Figure 2). The practice of eating raw meat and the close contact between humans and animals are both risk factors for the initiation of a new human CoV outbreak. This is due to the constant exposure of humans in these cultures to the ever-changing mutant CoVs.

The first cases of SARS-CoV-2 infections were reported in the Chinese city of Wuhan during December 2019 [11]. Although other options have not been completely ruled out yet, it is believed that the SARS-CoV-2 stemmed from a large seafood and animal market in Wuhan, the capital of Hubei province, China [11]. As for other wet markets in China, live animals are sold, mostly for food or medicine. The attributed medicinal and/or magical uses of wildlife and rare animal parts (such as pangolin scales and tiger paws) are mainly based on Traditional Chinese Medicine (TCM), which has been widely promoted by the current Chinese government. However, it is important to note that most of these folk remedies are never prescribed in reputable TCM hospitals. The Wuhan market is known to have a lot of exotic animals and exotic animal parts [42]. Thus, SARS-CoV-2 disease can be considered a zoonotic disease (like SARS) that has initially spread from animals to humans. However, human-to-human transmission has also been confirmed [79]. It is not well understood why outbreaks of CoV infections are mostly occurring in China. We speculate that those viruses may be predominantly circulating in animals in China rather than other animals in different parts of the world. One of the reasons for these sudden outbreaks could be the close interactions with live and wild animals that are consumed as food in wholesale food markets in China.

In a very recent study, the genomes of CoVs isolated from nine patients having viral pneumonia in Wuhan were analyzed [42]. The study showed that the genomes of these viruses differed by less than 0.1 percent (more than 99.98% of sequence identity), which indicates that the virus has only recently emerged in humans and has been detected rapidly after its emergence [42]. As the virus keeps spreading to more individuals, more mutations may arise which can potentially make the virus more virulent and thus constant surveillance will be necessary. The U.S. Center for Disease Control and Prevention (CDC) reported that SARS-CoV-2 causes a respiratory illness that is characterized by fever, cough, and shortness of breath. Radiographs of some SARS-CoV-2 patients demonstrated invasive lesions in both lungs [80]. The CDC also reports that the elderly, individuals with underlying health problems, and people with compromised immune systems are at a particularly higher risk of developing severe pneumonia from the virus [77].

We believe that it is too early to assess the impact of the new virus on children. SARS-CoV infections were significantly less common among children than adults, and kids younger than 12 reported much less severe symptoms than patients who were more than 12 [81]. This may have to do with children being exposed to more CoV in school and the outdoors than adults or because of the better overall health status of children as compared to adults. Also, children tend to be more up-to-date with vaccinations, which may protect them from secondary infections that are often triggered by the main infections.

During the SARS-CoV outbreak, most schools and factories in China remained open. Following the SARS-CoV-2 outbreak and confirmation of many infected cases, China responded by locking down residents of Wuhan city, banning wildlife trade until the epidemic is over, and attempting to build two new hospitals in the city of Wuhan to specifically handle the new outbreak. It is currently unclear if these two hospitals will be able to handle a major outbreak in a city of 11 million residents. There is no definite information about the exact time of the start of the outbreak. That is why it is hard to assess how contagious the virus is (the rate of sustained spread) at the present time. However, it seems likely and plausible that it is highly contagious, based on the mounting data about human-to-human transmission outside China [42]. In order to be able to precisely assess the rate of sustained spread, information about numbers of cases and deaths (the overall number of patients) will need to be divided by the overall number of people at risk of acquiring the disease (the number of individuals who have been in contact with the patients). Once this is determined, the overall risk can be assessed. Underreporting or misdiagnosis of cases can negatively impact the calculations and thus delay the ability of public health officials to truly assess the situation.

In an outbreak of this magnitude, the most important number that public health experts will be looking for is the basic reproduction number, also known as the R0 [82]. This number measures the disease’s potential and represents the average number of people who will catch the disease from one infected person in a population that has never had the disease in the past [82]. In one study, the mean estimate of R0 was calculated to be between 2.24 and 3.58 [83]. Another study estimated the R0 to have a high average value of 2.5 [84], which is consistent with other groups that reported values from 2 to 3 [83,85,86,87,88]. These R0 estimates for the SARS-CoV-2 are consistent with R0 estimates for SARS- and MERS-CoVs (from 2 to 5) (Table 1) [89,90]. The virus is less deadly than SARS, which killed about 10% of the infected patients. Because R0 represents an average, outliers (carriers who infect so many people or carriers who infect nobody) can have a huge impact on the final value of the R0. In other words, a bigger R0 does not necessarily mean more infections. For example, the R0 for the seasonal flu typically ranges from 1.2 to 1.4 [91], but it still infected many more people than the number of people who got infected with SARS-CoV. At any rate, any R0 above 1 should be taken seriously. The goal will be to reduce the R0 to a value that is below 1. Finally, R0 estimates can be higher than the “true” R0 values because of two main reasons: infected people who did not show symptoms and infected people who did not report their symptoms. Since R0 is not an intrinsic property of the virus itself, R0 estimates tend to be lower in places where there are sound infection control methods and vice versa. Typically, R0 estimates are highest at the beginning of an outbreak and then subside gradually once countries become aware of the outbreak and manage to put in effective control measures to prevent the spread of the virus.

The lockdown strategy that China is implementing works best during the early stages of an infection, which is not the case in Wuhan, as there are several million people who already left the city before the restrictions were imposed. If we are beyond the early stages, then there can be disadvantages associated with such lockdown on Wuhan. Among the disadvantages are people evading care to avoid any restrictions on their life.

Given that the incubation period can be up to 11 days or more, a two-week federal quarantine was ordered for 195 U.S. citizens who were flown back from China [92]. The action was described by the CDC as precautionary and preventive. For comparison, there were no quarantines ordered in the U.S. for the recent SARS-CoV or MERS-CoV outbreaks. The last federal quarantine in the U.S. was ordered back in the 1963 to prevent the spread of smallpox from Sweden to the U.S. during a smallpox outbreak in Sweden [93].

## 7. Protective Measures to Prevent Spread of the Disease

As shown in Table 1, the number of people infected by the virus has exceeded the global total infected with SARS-CoV (8098 individuals) in a nine-month period that extended from 2002 until 2003. It will be difficult to control the disease without a level of disruption of air travel. We believe that limiting travel to and from China will be a necessary measure.

Respiratory viruses spread through respiratory droplets that are produced when an infected person coughs or sneezes. The exact modes of transmission of SARS-CoV-2 are not entirely clear at this early stage, but reports of healthcare professionals in China who contracted the disease suggest a highly contagious virus [77]. Prevention of the SARS-CoV-2 infections entail precautions that are common to other respiratory viruses. The most obvious measure is to avoid contact with people who are sick. This is especially important due to the contagious nature of the virus. If someone is sick, they should stay home. Once they recover, they may consider using disposable face masks (while frequently changing them) and avoiding close contact with coworkers. However, the value of wearing face masks is controversial, to say the least [94]. Surgical masks do not fully protect against airborne viruses as they do not fully seal the nose and the mouth. Thus, small droplets, which can travel farther than large droplets and in more unpredictable patterns, can be inhaled around the sides of the masks. The N95 masks offer a better protection as long as they fit properly. It is worth noting that N95 masks are not suitable for people with facial hair [95].

Being an enveloped virus, washing hands with water and soap for at least 30 s would be beneficial in deactivating the SARS-CoV-2 [96]. Hand sanitizers can also be used if water and soap are not readily available, while touching eyes, nose, and mouth should be prevented [97].

Disinfection of different environmental surfaces, tools, and objects is crucial in limiting the spread of the virus [97]. The more you use one of these objects or touch one of these surfaces, the more pressing cleaning and disinfection become.

Public health officials, local health departments, hospitals, doctors, and CDC personnel should work closely with universities and other workplaces to educate and provide needed supplies to contain the spread of the virus. The World Health Organization (WHO) declared SARS-CoV-2 as a public health emergency of international concern (PHEIC) on 30 January 2020 [98]. A PHEIC is an atypical event that constitutes a public health risk and potential for the disease to spread to other countries, thus requiring a coordinated international response [99]. This designation could help mobilize more resources to the impacted areas. The current outbreak represents the sixth time the WHO has declared a global emergency since it gained the power to declare an international emergency in 2005 [100]. The previous five times were the 2009 H1N1 swine flu, the 2013 Ebola outbreak in West Africa, the 2014 polio outbreak, the 2016 Zika outbreak, and 2019 Ebola outbreak in the Democratic Republic of Congo [100]. None of these previous emergencies led a worldwide pandemic. However, we predict that the current outbreak is more likely to become a pandemic.

The current diagnostic test for the virus is PCR-based [101]. Since this test typically takes 48 h, a new quicker diagnostic test needs to be developed. It will not be practical to isolate (quarantine) a large number of individuals until results of the PCR-based diagnostic test become available. This can also overwhelm healthcare facilities, already overwhelmed by the increasing number of cases that are discovered every day in China and elsewhere in the world.

Containing the outbreak before it can spread is the best way to prevent pandemics. Border closures and screening at airports and checkpoints are classical measures that were previously implemented in the 2009 H1N1 flu pandemic [102]. This can reduce the spread of the virus but will not be a fool-proof strategy. The reason is that the incubation period of the virus is believed to be as long as 14 days, as was the case with MERS-CoV [103,104]. This means that carriers of the virus can show up at the border with no apparent symptoms and readily pass through security without raising any red flags. Compared to the SARS-CoV outbreak in 2003, the current increase in air traffic in China and worldwide has likely contributed to the more rapid spread of SARS-CoV-2 in 2020.

Without a quick diagnostic test, there are not many good options that can completely stop the transmission of the virus. Once a test is available, cases can be identified and isolated. Based on previous genetic experience with SARS-CoV, scientists will need to quickly develop a vaccine for SARS-CoV-2.

The world is hoping to succeed in containing this virus as soon as possible. Even after success, there needs to be follow-up with patients who are cured and declared virus-free. This is a lesson we learned from the Ebola outbreak, in which some patients who walked out from the hospital “virus-free” were found later to harbor the Ebola virus that was carefully hiding itself in other parts of the body, such as the immune-privileged eye [105,106,107]. Although this hidden Ebola virus was no longer transmissible to other humans, it rendered the label “virus-free” incorrect with these individuals.

## 8. Concluding Remarks

Natural disasters bring people together but epidemics and outbreaks split them apart. The SARS-CoV-2 is another CoV that may lead to a pandemic, if not timely controlled. Our current knowledge of this virus suggests an intermediate host; however, human-to-human transmission is confirmed and is of concern. The number of infected cases to date indicates a very rapid and efficient human-to-human transmission. This necessitates quick development of therapeutics that can inhibit this viral infection. Neutralizing antibodies and vaccines could play significant roles in controlling the SARS-CoV-2 outbreak.

## Figures and Tables

**Table 1:** Comparison between SARS-CoV, MERS-CoV, and the SARS-CoV-2, with respect to receptor usage, primary and intermediate hosts, incubation period, number of cases and deaths, and basic reproduction number (R0).

Virus	Receptor	Primary Host	Intermediate Host	Incubation Period	Number of Cases	Number of Deaths	Fatality	R0
**SARS-CoV**	ACE2	Bats	Civets and raccoon dogs	Typically between 2 and 10 days, and up to 14 days	8098	774	≈ 10%	2–5
**MERS-CoV**	DPP4 (CD26)	Bats	Camels	Typically between 2 and 14 days	2506	862	≈ 35%	2–5
**SARS-CoV-2**	Most probably ACE2	Most probably Bats	Not identified	Current estimates between 2 and 10 days, and up to 14 days	Over 92,000 as of 3 March 2020	Over 3000 as of 3 March 2020	3.4% as of 3 March 2020	2–3.5
